# Randomised controlled trials evaluating anticancer therapies in haematological cancers: an overview of global research activity

**DOI:** 10.3332/ecancer.2023.1558

**Published:** 2023-06-08

**Authors:** Manju Sengar, Wilma M Hopman, Ghulam Rehman Mohyuddin, Aaron M Goodman, Bishal Gyawali, Deborah Mukherji, Nazik Hammad, CS Pramesh, Ajay Aggarwal, Richard Sullivan, Christopher M Booth

**Affiliations:** 1 Tata Memorial Centre, Homi Bhabha National Institute, Mumbai 400012, India; 2 Department of Public Health Sciences, Queen’s University, Kingston, ON K7L 3N6, Canada; 3 Division of Hematology and Haematological Malignancies, University of Utah, Salt Lake City, UT 84112, USA; 4 Division of Blood and Marrow Transplantation, University of California, San Diego, CA 92093, USA; 5 Department of Oncology, Queen’s University, Kingston, ON K7L 5P9, Canada; 6 Division of Cancer Care and Epidemiology, Queen’s University Cancer Research Institute, Kingston, ON K7L 3N6, Canada; 7 American University of Beirut Medical Center, Beirut 11-0236, Lebanon; 8 Institute of Cancer Policy, King’s College London, WC2R 2LS London, UK; 9 London School of Hygiene and Tropical Medicine, WC1E 7HT London, UK

**Keywords:** randomised controlled trials, haematological cancers, endpoints

## Abstract

**Background:**

Design, results, and interpretation of oncology randomised controlled trials (RCTs) have changed substantially over the past decade. In this study, we describe all RCTs evaluating anticancer therapies in haematological cancers published globally during 2014–2017 with comparisons with solid tumours RCTs.

**Methods:**

A PubMed literature search identified all phase 3 RCTs of anticancer therapy for haematological cancers and solid tumours published globally during 2014–2017. Descriptive statistics, chi-square tests and the Kruskal–Wallis test were used to compare RCT design results, and output between haematological cancers and solid tumours as well as for different haematological cancer subtypes.

**Results:**

694 RCTs were identified; 124 in haematological cancers and 570 in solid tumours. Overall survival (OS) was the primary endpoint in only 12% (15/124) of haematological cancer trials compared to 35% (200/570) in solid tumours (*p* < 0.001). Haematological cancer RCTs evaluated the systemic novel therapy more often than the solid tumour RCT (98% versus 84%, *p* = 0.002). Use of surrogate endpoints like progression-free survival (PFS) and time to treatment failure (TTF) were more common in haematological cancers than solid tumours (47% versus 31%, *p* < 0.001). Within haematological cancers, the use of PFS and TTF was more prevalent in chronic lymphocytic leukaemia and multiple myeloma as compared to others (80%–81% versus 0%–41%, *p* < 0.001). Seventy-eight percent of haematologic trials were funded by industry as compared to 70% of solid tumour trials. Only 4% (5/124) of haematologicalcancer trials were led by investigators in upper-middle and lower-middle-income countries as compared to the 9% of solid tumour trials.

**Conclusion:**

The fact that only 12% of haematological cancer RCTs are designed to show improvements in OS is of grave concern for the field and the care of future patients. This is further compounded by the highly prevalent use of alternative primary endpoints that are rarely valid surrogates for OS in haematological cancers.

## Introduction

The introduction of effective therapeutics and supportive care have led to substantial improvements in outcomes for common haematological cancers, particularly for chronic myeloid leukaemia (CML), Hodgkin lymphoma, diffuse large B-cell lymphoma, and multiple myeloma (MM) [1, 2]. Much of this progress comes from new medicines introduced in the early 2000s based on the results of phase III randomised controlled trials (RCTs) [1]. While progress continues to be made, there is growing concern that the clinical trial landscape for haematological cancers is dominated by drug approvals based on single-arm trials [3, 4]; Even among medicines that are tested in RCTs, there are concerns about the use of surrogate endpoints which are not validated and inadequate control arms. For patients with cancer irrespective of the type of cancer, the most important outcomes are overall survival (OS) and quality of life (QoL). Any other RCT endpoints should be valid surrogates for OS or QOL. Unfortunately, in most cancers, the level of surrogacy is poor [4, 5]. There is also growing recognition that many new treatments for cancer are associated with very small effect sizes [6, 7]. Adoption of cancer treatments with uncertain and/or small benefits poses risks to patients and the health system with substantial clinical, financial and time toxicities [8, 9]. This is a major concern given the rising burden of haematological cancers, more so in resource-constrained systems which are already facing challenges to deliver high-quality care [10].

Bibliometric analyses of published RCTs in solid tumours have identified important temporal trends, including a major shift towards industry funding of palliative intent drug trials. The use of OS as the primary endpoint has decreased over time and is now replaced by progression-free survival (PFS) as the most common endpoint in oncology [11]. Recent work by Wesson *et al* [12] identified some worrisome trends among active clinical trials in haematological cancer. Using information from ClinicalTrials.gov, Wesson *et al* [12] identified 2,609 trials registered during 2015–2020. Among the 2,609 trials included in this cohort, only 21% (538/2,609) were randomised. Moreover, this proportion decreased over the study period from 26% in 2015 to 19% in 2020 (*p* < 0.001). Their other striking finding was the observation that among RCTs, only 15% used OS or QOL as the primary endpoint. The remaining 85% of trials tested alternative endpoints, most commonly tumour response (235/538, 44%) and PFS/relapse-free survival (205/538, 38%). The study was limited in its scope as it did not include published results of trials. To address this knowledge gap, we undertook the following study to describe the methodology and results of all haematological cancer RCTs published globally during 2014–2017. For comparative purposes, we contrast these findings with RCTs of a solid tumour during the same time period.

## Methods

### Study design and search strategy

This study is a secondary analysis of a recently published retrospective cohort study of global oncology RCTs published between 2014 and 2017. The original study design and cohort generation are described elsewhere [13]. A structured PubMed literature search used the following terms: *Neoplasms/drug therapy* (MeSH) OR *Neoplasms/radiotherapy*(MeSH) OR *Neoplasms/surgery* (MeSH) OR *Neoplasms/therapy* (MeSH) OR *Neoplasms/transplantation* (MeSH), sorted by best match and filtering for phase 3 clinical trials.

Studies were included if they were English-language reports with a phase III clinical trial design. Subset/pooled analyses and reports of the interim analyses were excluded. The primary objective of the study is to evaluate differences between solid and haematological malignancies with respect to the proportion of trials using OS and QoL as primary endpoints. The secondary objectives were to evaluate differences between solid and haematological malignancies in terms of frequency and pattern of surrogate endpoint use, sample size, magnitude of benefit in the studies which met their primary endpoint, representation of low-middle income countries in the trials, and the impact-factor (IF) of journals which published positive and negative studies.

### Data abstraction and classification

Data collection was performed independently by two individuals using a standardised collection template. Variables included authorship, funding, disease subtype, study design, sample size, the primary endpoint of the study, the magnitude of benefit if the study met the primary endpoint, and the journal of publication. The funding source was identified from explicit statements in the manuscript or acknowledgment section. Studies were classified into the country of origin based on the institutional affiliation of the first author; the country of origin was used to further divide studies into lower-middle, upper-middle-income (UMIC/LMIC) or high-income countries (HICs) [14]. Journal IF was compared using the IF from 2016 as reported by the journal citation reports IF [15]. The senior author (C.M.B.) performed random data checks to ensure abstraction was of high quality. After data collection, a further 30 studies were randomly evaluated for concordance; only 11/1,020 (1%) variables were discordant with the original assessment.

### Statistical analysis

Statistical analysis was conducted using IBM SPSS version 27.0 for Windows (Armonk, New York, 2021). Descriptive results (frequencies, percentages, medians and quartiles) were generated for the full study cohort and by disease site. Comparisons were then made between disease-specific RCTs using the Pearson chi-square or Fisher’s exact test, and the Mann–Whitney *U* or the Kruskal–Wallis as appropriate. *p* values less than 0.05 were considered significant; no adjustments were made for multiple comparisons.

## Results

### Results of the search strategy

A total of 2,275 publications were identified, of which 694 were phase III RCTs evaluating anti-cancer therapy (Supplemental eFigure). Among these, 124 RCTs involved haematological cancers; acute leukaemias (*n* = 20), lymphomas (*n* = 42), chronic lymphocytic leukaemia (CLL) (*n* = 20), MM (*n* = 31), CML (*n* = 8) and myelodysplastic syndrome/myeloproliferative neoplasms (MDS/MPN) (*n* = 3). The remaining 570 trials were trials on solid tumours.

### Setting and design of RCTs

Comparative design characteristics of haematologic and solid tumour trials are shown in Table 1. Details across specific haematologic diseases are shown in Table 2. Almost all (98%, 121/124) haematological cancer trials tested new systemic therapy compared to 84% (480/570) of solid tumour trials (*p* = 0.002). OS as the primary endpoint was substantially less common among haematological cancer trials compared to solid tumours (12% (15/124) versus 35% (200/570) *p* = <0.001). Trials for acute leukaemia used OS more often than other haematological cancers (45% versus range 0%–7%, *p* < 0.001). The use of surrogate endpoints like PFS and time to treatment failure (TTF) was more common in haematological cancers than solid tumours (47% (58/124) versus 31% (174/570), *p* < 0.001). Within haematological cancers, the use of PFS and TTF was more prevalent in CLL and MM as compared to others (80%–81% versus range 0%–41%, *p* < 0.001). The response rate was the primary endpoint for 16% and 4% of haematologic and solid tumour trials, respectively. Six of eight (75%) trials in CML used response rate (complete cytogenetic and major molecular response) as the primary endpoint.

Seventy-eight percent (97/124) of haematological cancer trials were funded by the industry compared to 70% (396/570) of solid tumour trials (*p* = 0.052). This proportion varied substantially across haematologic diseases, from 55% (11/20) in acute leukaemia to 90% (18/20) in CLL and 100% in CML (8/8) and MDS/MPN (3/3) (*p* = 0.042). Ninety-six percent (119/124) of haematological cancer RCTs and 91% (517/570) of solid tumour trials were led by HICs. The top six countries within HIC were North America, Germany, France, Italy, the United Kingdom and Canada.

### Sample size and outcome of RCTs

Haematological cancer RCTs were substantially smaller (median sample size 373 (IQR-236–544) versus 465 (250–800), *p* = 0.001) than solid tumour trials and more likely to meet their primary endpoint (56% (59/124) versus 45% (256/570), *p* = 0.037). The observed effect size among positive trials was similar for haematological cancers and solid tumours (HR 0.67 and HR 0.69, respectively). The median sample size for MM trials (498) was larger than the other haematological cancers (acute leukaemias 349, lymphoma 363, CLL 298, CML 287, MDS/MPN 149). The proportion of haematological cancer trials which met their primary endpoint ranged from 40% in acute leukaemia to 68% in MM.

**Figure 1.**
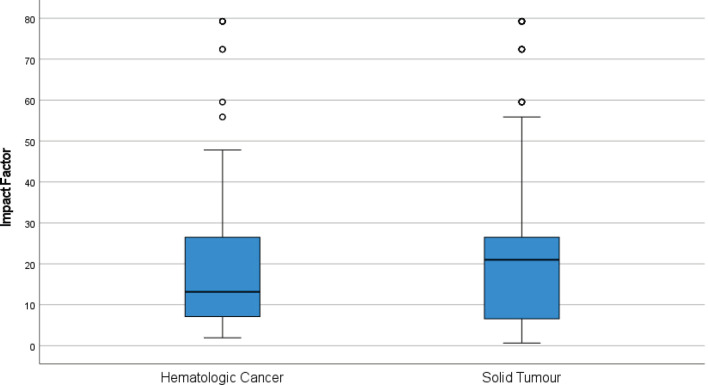
Differences in journal IFs between hematological cancer (*n* = 124) and solid tumour (*n* = 570) RCTs published 2014–2017. Circles and asterisks within the figures represent outliers and far (more extreme) outliers respectively.

**Figure 2.**
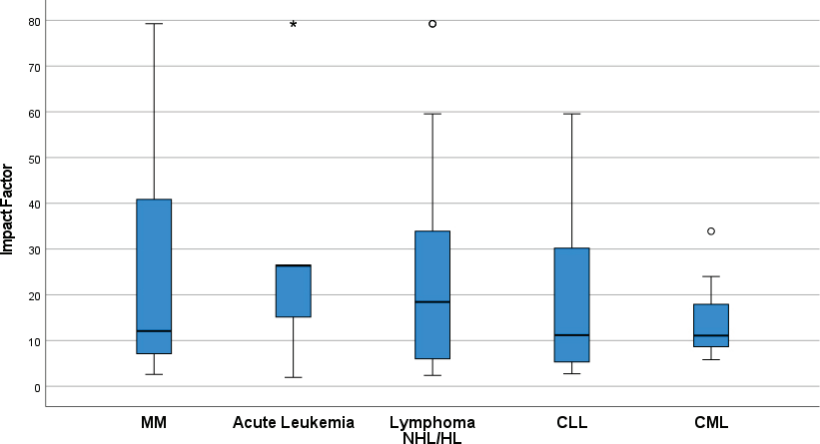
Differences in journal IFs across RCTs of different hematological cancers published 2014–2017. (CLL-Chronic lymphocytic leukemia, CML -Chronic myeloid leukemia, HL- Hodgkin lymphoma, MM- Multiple myeloma, NHL- Non Hodgkin lymphoma). Circles and asterisks within the figures represent outliers and far (more extreme) outliers respectively.

Ninety-nine percent (686/694) of RCTs were published in journals with IF. The median IF for all RCTs was 18 (IQR 7–27). Overall the IF for journals in which haematological cancer trials were published was lower than solid tumours (13 versus 21, *p* = 0.768); stratified analyses by trial outcome show this was largely driven by ‘negative’ haematological cancer RCTs being published in lower impact journals than negative RCTs of solid tumours (Figure 1). The IF across haematological cancers is shown in Figure 2.

## Discussion

In this study, we describe key features of contemporary RCTs in haematological cancers and contrast them with those in solid tumours. Several important findings have emerged. First – and perhaps of greatest concern – is the observation that only 12% of haematological cancer RCTs use OS as the primary endpoint. Second, across haematological cancers, there was substantial variation in the use of alternative endpoints PFS/TTF; ~80% of trials in MM/CLL are now using these endpoints. Third, the vast majority of haematological cancer trials are funded by the pharmaceutical industry. Fourth, the proportion of haematological cancer trials that met their primary endpoint varied substantially across diseases, from 55% in acute leukaemia to 90%–100% in CLL, CML, and MDS/MPN. Finally, only a handful of haematological cancer RCTs are led by investigators in UMIC/LMIC – this is a major problem given the massive increase in disease burden being observed in these emerging economies [16].

Haematological cancer clinical trials fall short on both parameters, which are most relevant for patients – living longer (OS) and/or living better (QoL). Only 12% of haematological cancer trials used OS as the primary endpoint in comparison to 35% of solid tumour trials. The longer survival of some haematological cancers may explain this observation, although it is not necessarily an adequate justification. Trials in acute leukaemia fared better with regard to the use of OS as the primary endpoint, given the relatively shorter OS in this subgroup of patients. Use of QoL or toxicity as the primary endpoint was observed in only 3% of trials, limited to acute leukaemia and lymphoma. However, given the continuous nature of several novel therapies in CLL and MM, it may be a more relevant endpoint in these cancers [17, 18].

The use of surrogate endpoints was much more common for haematological cancer trials as compared to solid tumour RCTs. PFS/TTF were common endpoints in MM and CLL trials. Although widely accepted by regulatory authorities, PFS or TTF has not been shown to be a consistent surrogate for OS in MM [19], with some trials having demonstrated worse OS despite improvement in PFS [20, 21]. Similarly, there is no evidence to suggest that improvement in PFS is associated with improved QoL [22]. These endpoints need to be interpreted even more carefully when there is an imbalance due to a weaker control arm or duration of therapy, a common occurrence in trials of MM and CLL [3, 23, 24]. The use of these surrogate endpoints with flawed control arms may lead to an early approval of an ineffective and toxic therapy as seen with melflufen [25]. Trials in CML used response rates such as rates of complete cytogenetic and major molecular response for evaluating the second-generation tyrosine kinase inhibitors. These trials did meet their primary endpoint without an impact on either PFS or OS as compared to imatinib, raising the concern of toxicities like cardiovascular events, which can compromise OS and QoL [26, 27].

The study highlights the growing trend of industry funding clinical trials. A higher proportion of haematological cancer RCTs (78%) received industry support. Industry partnership is crucial in drug development and potentially changing the treatment paradigm. However, unfortunately, it increases the possibility of selecting a less robust study design, endpoints, assessments, emphasis on statistical significance over clinical significance, and unplanned analyses, which may lead to drug approval [28]. This further reduces the opportunity for studies addressing drug repurposing, toxicities, comparing the two standard interventions for a disease (comparative effectiveness research, and integration of palliative care – these questions matter greatly to patients but are of less interest to the pharmaceutical industry [29, 30].

The epidemiologic burden of haematological cancer varies substantially across the globe. However, despite having a lower incidence, given the large population of many UMIC/LMICs, haematological cancers still represent a substantial public health burden. Accordingly, it is a major gap that only a handful of RCTs are led by investigators in UMIC/LMIC. This likely reflects limited funding opportunities and research infrastructure capacity to lead RCTs. Bi-directional collaborations and new sources of funding are needed to remedy this imbalance. Ironically, the drugs which get approved based on RCTs from HIC are eventually marketed in UMIC/LMIC. However, not including patients from these health systems leads to the risk of increased toxicity/differing outcomes based on differences that may exist in population-level pharmacokinetics and pharmacodynamics. Except for biosimilar research and phase IV trials, it is rare for industry-sponsored research to be conducted primarily in LMICs for drug registration purposes. These practices further widen the access gap for the effective therapies [31, 32].

It is useful to consider our results in light of the recent analysis by Wesson *et al* [12] which evaluated all trials registered on clinicaltrials.gov. The use of clinically relevant endpoints (OS and QoL) was similar in both of these studies (15%). In contrast to our study, Wesson *et al* [12] reported a higher representation of UMICs/LMICs-led trials (17% versus 4%). However, the methodology of what was considered a UMIC/LMIC trial varies between our studies. Wesson *et al* [12] designated a trial as LMIC if it were run exclusively in one or more LMIC, but if the trial ran in a UMIC or HIC, it was not designated as an LMIC trial. This varies from our method as classified into LMIC or HIC based on the institutional affiliation of the first author. Furthermore, Wesson’s analysis used data from trial registration only, and it would be important to see how many of the trials from UMIC/LMIC faced funding challenges and were terminated prematurely.

Our study results should be interpreted in light of methodological limitations. While our study provides a broad overview of all the studies but it included a relatively short time frame of 2014–2017. In addition, we did not evaluate individual studies in depth to understand the appropriateness of the control arms, absolute magnitude of benefit, assumptions for sample size calculation, secondary endpoints, and the implications on approval of drugs. Our study did not evaluate one of the big threats to drug approvals based on single-arm early-phase trials. There are several examples of such approvals- acalabrutinib (*r*/*r* mantle cell lymphoma), ivosidenib (*r*/*r* IDH mutated acute myeloid leukaemia), zanubrutinib (*r*/*r* CLL), belantamab mafodotin (*r*/*r* MM), CAR- T cell therapies [33–39, 40]. The literature search in our study was restricted to RCTs reported in PUBMED; trials that were conducted but not published were not included in our search strategy. Previous research has shown that a substantial proportion of conducted trials are never published and that the unpublished trials may be different than the ones that are published. As such, this could skew our analysis [41, 42]. However, these limitations are beyond our control as it would be difficult, if not impossible to get raw or analysed data from unpublished trials.

## Conclusion

In summary, this study offers important insights into the state of clinical trials in haematological cancers. The fact that only 12% of haematological cancer RCTs are designed to show improvements in OS is of grave concern for the field and the care of future patients. The field must confront the striking shift towards alternative endpoints. All attempts should be made to assess OS, either as a primary endpoint or as a study follow-up which can enable the validation of surrogate endpoints against the OS. The emphasis should be to assess larger and more meaningful differences as compared to the comparator, use of appropriate comparator arm, and have a wider representation from LMIC in the design and execution of the study to ensure implementation across the resource settings.

## Funding

None.

## Conflicts of interest

**Manju Sengar, Wilma M. Hopman, Ghulam Rehman Mohyuddin, Nazik Hammad, CS Pramesh, Ajay Aggarwal, Richard Sullivan, Christopher M. Booth**- None

**Aaron Goodman:** Consultant and speaker for Seattle Genetics and EUSA Pharma

**Bishal Gyawali**: Consulting fees from Vivio Health, unrelated to the manuscript

**Deborah Mukherjee:** Astellas, Invited Speaker, Personal**,** Astra Zeneca, Invited Speaker, Personal**,** Bayer, Invited Speaker, Personal**,** BMS, Invited Speaker, Personal**,** Janssen, Invited Speaker, Personal**,** MSD, Invited Speaker, Personal**,** Pfizer, Advisory Board, Personal**,** Astellas, Research Grant, Institutional, No financial interest**,** BMS, Research Grant, Institutional, No financial interest**,** Janssen, Research Grant, Institutional, No financial interest**,** Novartis, Research Grant, Institutional, No financial interest.

## Contributors

MS, CMB and WM designed the study, collected and analysed the data, and wrote the first draft of the manuscript. WM wrote the statistical analysis plan. GRM, AM, CSP, AA, RS, NH, DM and BG provided critical inputs and reviewed the final draft. All the authors had full access to the data and had final responsibility for the decision to submit it for publication.

## Data sharing statement

The data will be made available by the corresponding author to the investigators interested in data sharing and collaboration.

## Figures and Tables

**Supplemental eFigure.**
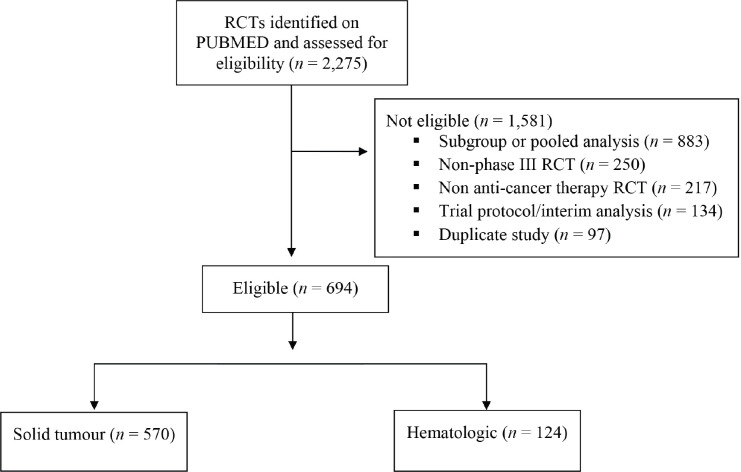
Results of search strategy for all oncology RCTs conducted during 2014–2017.

**Table 1.** Design characteristics and results of phase 3 RCTs in hematological and solid tumours published globally 2014–2017.

	All RCTs *n* = 694	Hematological *n* = 124	Solid tumour *n* = 570	*p*-value
	*N* (%)	*N* (%)	*N* (%)	
Experimental arm				
Systemic	601 (87)	121 (98)	480 (84)	0.002
Radiation	38 (6)	1 (1)	37 (7)	
Surgery	16 (2)	0 (0)	16 (3)	
Other/combinations	39 (6)	2 (2)	37 (7)	
Study design				
Superiority	610 (88)	109 (88)	501 (88)	1.00
NI/equivalence	84 (12)	12 (12)	69 (12)	
Primary endpoint				
OS	215 (31)	15 (12)	200 (35)	<0.001
DFS/EFS/RFS	149 (22)	24 (19)	125 (22)	
PFS/TTF	232 (33)	58 (47)	174 (31)	
Other^@^	98 (14)	27 (22)	71 (13)	
Country of origin^~^				
HIC	636 (92)	119 (96)	517 (91)	0.113
UMIC	51 (7)	5 (4)	46 (8)	
LMIC	7 (1)	0 (0)	7 (1)	
Industry funding	493 (71)	97 (78)	396 (70)	0.052
Total sample size				
Median (IQR)	443 (246–718)	373 (236–544)	465 (250–800)	0.001
Primary endpoint met				
Yes	325 (47)	69 (56)	256 (45)	0.037
No	369 (53)	55 (44)	314 (55)	
^ IF	*n* = 686	*n* = 124	*n* = 562	
Median (IQR)	21 (7–27)	13 (7–27)	21 (7–28)	0.768
IF for + RCTs	*n* = 318	*n* = 69	*n* = 249	
Median (IQR)	25 (8–44)	25 (11–48)	25 (7–36)	0.398
IF for − RCTs	*n* = 368	*n* = 55	*n* = 313	
Median (IQR)	18 (6–26)	12 (5–24)	18 (6–26)	0.024
HR for + RCTs	*n* = 217^*^	*n* = 49^a^	*n* = 168^b^	
Median (IQR)	0.68 (0.64–0.74)	0.67 (0.61–0.72)	0.69 (0.64–0.75)	0.161

RCTs = randomized controlled trial, OS = overall survival, DFS = disease-free survival, EFS = eventfree survival, RFS = relapse-freeFS = progression free survival, TTF = time to treatment failure, NI = non-inferiority, HIC = High-income country, UMIC = upper-middle income country, LMIC = low-middle-income country. QOL = quality of life

* Treatment intent was reported as ‘other’ for one and ‘not stated for another’, therefore *n* = 692

@ Other primary endpoints include QOL/toxicity (*n* = 4 and 17), response rate (*n* = 20 and 24), other or not specified (*n* = 3 and 30) for hematological and solid tumours, respectively

~ Based on the institutional affiliation of the first author

^ Impact factor not available for 6

Note that percentages may total to 101 due to rounding

*p*-values are based on the Fisher’s Exact test

RCT = Randomized controlled trial; IQR = interquartile range; HR = hazard ratio

^*^Only reported for *n* = 262 positive superiority trials but HR unavailable for 45, *n* = 217

a Only reported for *n* = 59 positive superiority trials, but HR unavailable for 10, *n* = 49

b Only reported for *n* = 203 positive superiority trials but HR unavailable for 35, *n* = 168

*p*-values are based on the Fisher’s exact test or the Mann-Witney U

**Table 2.** Design and results of all RCTs in hematological cancer published during 2014–2017.

All RCTs *n* = 124	MM *n* = 31	Acute leukemia *n* = 20	Lymphoma NHL/HL *n* = 42	CLL *n* = 20	CML *n* = 8	MDS/MPN *n* = 3	*p*-value	
	*N* (%)							
Primary endpoint OS DFS/EFS/RFS PFS/TTF QOL/toxicity RR Other Industry funding Yes No Sample size Median (IQR) IF Median (IQR) Primary EP met Yes No	15 (12) 24 (19) 58 (47) 4 (3) 20 (16) 3 (2) 97 (78) 27 (22) 373 (236, 544) 13 (7–27) 69 (56) 55 (44)	2 (7) 1 (3) 25 (81) 0 (0) 3 (10) 0 (0) 23 (74) 8 (26) 498 (271, 668) 12 (7–48) 21 (68) 10 (32)	9 (45) 7 (35) 0 (0) 2 (10) 2 (10) 0 (0) 11 (55) 9 (45) 349 (243, 535) 26 (13–27) 8 (40) 12 (60)	3 (7) 13 (31) 17 (41) 2 (5) 5 (12) 2 (5) 34 (81) 8 (19) 363 (223, 531) 18 (6–34) 21 (50) 21 (50)	1 (5) 0 (0) 16 (80) 0 (0) 3 (15) 0 (0) 18 (90) 2 (10) 298 (230, 468) 11 (4–32) 13 (65) 7 (35)	0 (0) 2 (25) 0 (0) 0 (0) 6 (75) 0 (0) 8 (100) 0 (0) 287 (216, 515) 11 (8–21) 4 (50) 4 (50)	0 (0) 1 (33) 0 (0) 0 (0) 1 (33) 1 (33) 3 (100) 0 (0) 149 (129, n/a) 26 (8, n/a) 2 (67) 1 (33)	<0.001 0.042 0.201 0.499 0.391

Percentages may not total 100 due to rounding; *p*-values are based on the Fisher’s exact test or the Kruskal-Wallis test

RCTs = randomized controlled trial, OS = overall survival, DFS = disease free survival, EFS = event free survival, RFS = relapse free survival, PFS = progression free survival, TTF = time to treatment failure; QOL = quality of life; RR = response rate; IQR = inter-quartile range; EP = endpoint; MM = multiple myeloma; NHL = Non Hodgkin lymphoma; HL = Hodgkin lymphoma; CLL = chronic lymphocytic leukemia; CML = chronic myeloid leukemia;

MDS = myelodysplastic syndromes; MPN = myeloproliferative neoplasm
